# Effectiveness of a blended online behavioral parent training program for Vietnamese parents of children with ADHD: A randomized controlled trial with 3‐month follow‐up

**DOI:** 10.1002/jcv2.70150

**Published:** 2026-07-24

**Authors:** Tran Thanh Nam, Nguyen Thi Tram Anh, Nguyen Thi Hong Nhung, Le Thi Lam, Le Thi Thuy Hang

**Affiliations:** ^1^ Faculty of Education Sciences University of Education VNU Hanoi Vietnam; ^2^ Faculty of Psychology Education and Social Work The University of Danang – University of Science and Education Da Nang Vietnam; ^3^ Family Da Nang Psychological Research and Consulting Company Da Nang Vietnam

**Keywords:** ADHD, behavioral parent training, cultural adaptation, parenting skills, randomized controlled trial, Vietnam

## Abstract

**Background:**

Behavioral parent training (BPT) is an evidence‐based intervention for children with attention‐deficit/hyperactivity disorder (ADHD); however, evidence from low‐ and middle‐income Asian countries remains scarce. This randomized controlled trial evaluated a culturally adapted blended BPT program for Vietnamese parents of elementary school‐aged children with ADHD.

**Methods:**

Eighty‐six parents of children (6–11 years) with a clinician‐confirmed DSM‐5 diagnosis of ADHD were randomized to a blended BPT program (*n* = 43) or treatment as usual plus psychoeducational materials (*n* = 43). The intervention comprised six weekly 2‐h online group sessions, each followed within the same week by a 1‐h individual home‐based support session, and 12 weekly support telephone calls across a 3‐month maintenance period. Parenting skills (Parenting Skills Assessment Scale), parent‐rated child ADHD symptoms (Vanderbilt ADHD Diagnostic Parent Rating Scale), and parenting stress were assessed at baseline (T1), post‐intervention (T2), and 3‐month follow‐up (T3). The analyses included 80 participants with complete data (intervention, *n* = 37; control, *n* = 43).

**Results:**

Parenting skills improved substantially in the intervention group relative to the control group (group × time interaction: *F* (2, 156) = 20.75, *p* < .001, partial *η* ^*2*^ = 0.21; baseline‐adjusted between‐group *d* = 1.28 at follow‐up). Parent‐rated ADHD symptoms decreased significantly within the intervention group (T1→T3: −4.14 points, *p* < .001), but the baseline‐adjusted between‐group differences were small and not statistically significant (T2: *p* = .057; T3: *p* = .075; adjusted *d* ≈ 0.28–0.30). Parenting stress declined similarly in both groups. Exploratory mediation analyses indicated a significant indirect effect on follow‐up ADHD symptoms through improved parenting skills (*ab* = −1.86, 95% bootstrap CI [−4.01, −0.37]).

**Conclusion:**

The culturally adapted blended BPT program was feasible and produced large, sustained improvements in parenting skills among Vietnamese parents. The effects on parent‐rated ADHD symptoms were small and require confirmation in adequately powered trials.

**Trial registration:**

Thai Clinical Trials Registry, TCTR20260212003 (retrospectively registered on February 12, 2026). Registration number: TCTR20260212003. Available at: https://www.thaiclinicaltrials.org/show/TCTR20260212003.

## Key Points

- What is known?
  - Behavioral parent training (BPT) improves parenting practices and child behavior in ADHD, but most trials come from Western, high‐income settings; evidence from Southeast Asia is very limited.
- What is new?
  - In the first randomized controlled trial (RCT) of a culturally adapted, blended (online group plus home‐based support) parent training program in Vietnam, parenting skills improved markedly and durably; between‐group effects on parent‐rated ADHD symptoms were small and non‐significant, and improved parenting skills mediated the intervention–symptom association in exploratory analyses.
- What is relevant?
  - Blended delivery—brief online group training with individualized home‐based coaching and telephone follow‐up—is feasible and acceptable in a lower‐resource Asian context and may scalably strengthen parenting skills; child‐outcome effects require confirmation in larger trials.

## INTRODUCTION

Approximately 5% of children worldwide meet the diagnostic criteria for attention‐deficit/hyperactivity disorder (ADHD); however, many affected children, particularly in low‐ and middle‐income countries, never access evidence‐based care (Sayal et al., 2018). ADHD is associated with academic underachievement, peer difficulties, elevated injury risk, and substantial economic burden (Faraone et al., 2021), and places considerable strain on families: parents of children with ADHD report markedly higher parenting stress than parents of typically developing children (Theule et al., 2013).

### Behavioral parent training for ADHD: The evidence base

Behavioral parent training is one of the best‐established psychosocial interventions for children with ADHD. Meta‐analytic evidence indicates that BPT produces robust small‐to‐medium improvements in parenting outcomes—positive parenting, negative parenting, and parenting sense of competence—that hold even when outcomes are rated by masked assessors (Daley et al., 2014; Dekkers et al., 2022). The effects on children's ADHD symptoms are more modest: parent‐rated symptom improvements are typically small to moderate, and the effects on masked ratings of core symptoms are limited, whereas the effects on parenting and child conduct problems are more consistent (Daley et al., 2014). Component‐level meta‐regression analyses suggest that techniques involving the manipulation of antecedents (e.g., clear rules and instructions) and reinforcement of desired behavior are key active ingredients, whereas a higher dose of stand‐alone psychoeducation is associated with weaker effects (Dekkers et al., 2022; Hornstra et al., 2023).

Parenting a child with ADHD requires different skills than parenting typically developing children. Because ADHD‐related behaviors are driven by neurodevelopmental differences in attention, inhibition, and reward processing, parents must learn to restructure antecedents (predictable routines, short and specific instructions, advance warnings for transitions), deliver immediate, frequent, and salient reinforcement, apply consistent and brief consequences (planned ignoring, time‐out), and prevent escalation cycles of coercive parent–child interaction (Barkley, 2006; Patterson, 1982). Generic parenting advice that relies on delayed consequences or lengthy verbal reasoning is often ineffective for children with ADHD; therefore, BPT programs explicitly train ADHD‐specific behavioral techniques (Dekkers et al., 2022).

### Delivery formats: Online and blended approaches

Traditionally, BPT has been delivered face‐to‐face, but digital delivery has expanded rapidly. A pilot randomized trial directly comparing face‐to‐face with online BPT for young children at risk of ADHD found comparable engagement and improvements in parental knowledge, implementation fidelity, and child behavior across formats, although parents rated the face‐to‐face format as more acceptable (DuPaul et al., 2018). Digital parenting interventions, such as the STEPS app, are currently being evaluated at scale for families awaiting ADHD assessment (Kostyrka‐Allchorne et al., 2022), and a pilot online mindfulness‐based program for parents of children with ADHD has reported reduced parental stress and improved parent–child interactions (Lo et al., 2024). Simultaneously, the literature highlights the challenges of fully self‐directed online formats, including lower engagement, higher attrition, and reduced opportunities for individualized feedback and skill rehearsal (DuPaul et al., 2018). Blended models that combine structured online content with individualized, person‐delivered supportare one proposed solution, and hybrid culturally adapted programs have shown effectiveness and high retention in East Asian routine care (Shimabukuro et al., 2024).

In the present study, an online group format combined with home‐based individual support was chosen for pragmatic reasons specific to the study region in Central Vietnam. The participants were dispersed across urban, rural, coastal, and mountainous districts. Many parents worked shifts or in agriculture, making it difficult for them to attend fixed weekend clinics. Widespread smartphone and Internet access has made synchronous online group delivery feasible while reducing travel costs and disruptions from adverse weather (e.g., typhoons and flooding).

### Mechanisms of change: Parenting skills as mediator

Theoretically, BPT is expected to change children's behavior *through* changes in parenting. Reviews of mediation studies of BPT for externalizing problems support changes in parenting practices as a plausible mechanism of change, while noting that formal mediation tests remain relatively scarce (Forehand et al., 2014). Most directly, a recent individual participant data meta‐analysis of 19 randomized trials in children with ADHD found that improvements in parenting behaviors and parent–child affection jointly explained concurrent improvements in children's ADHD severity, oppositional behavior, and functional impairment (Psyllou et al., 2025). Testing this mediational pathway in a Vietnamese sample addresses an open question: whether the parenting‐mediated mechanism documented in Western samples generalizes to a Confucian‐heritage collectivist family context. Therefore, we specified a priori that improvements in parenting skills would mediate any intervention effect on child ADHD symptoms (see the conceptual framework below).

We additionally measured parenting stress as a secondary outcome for two reasons. First, parenting stress is reliably elevated in parents of children with ADHD and is bidirectionally linked to child behavior and parenting practices (Theule et al., 2013). Behavioral parent training trials conducted in Asia have reported stress reduction as a primary benefit (Shimabukuro et al., 2024). Second, examining stress allows for a check on the specificity of the proposed mechanism: if intervention effects operate through parenting skills rather than general distress reduction, the indirect effect through skills should remain after accounting for parenting stress.

### The Vietnamese context and the need for cultural adaptation

Most BPT programs were developed in Western, individualistic cultural contexts. Vietnamese family life, shaped by Confucian traditions, differs in ways that are directly relevant to parent training. First, family hierarchy and filial piety are central: children are expected to obey, and overt parental praise of children is less normative, with some parents viewing frequent praise as risking “spoiling” the child (Mestechkina et al., 2014). Second, the cultural notion of *training* (similar to the Chinese *chiao shun*; Chao, 1994) frames strict, highly involved parenting as an expression of care, which can make techniques such as planned ignoring or child‐directed play, counterintuitive. Third, multigenerational households are common—in our sample, roughly one‐third of families lived with grandparents—so behavior plans require negotiation with co‐caregiving elders. Fourth, academic achievement carries exceptional weight, concentrating on parent–child conflict around homework. Finally, mental health literacy regarding ADHD is limited, and services are concentrated in large cities. International experience indicates that parent training programs can be successfully adapted across cultural contexts, but systematic adaptation of materials, examples, and delivery—with input from local stakeholders—is required (Shimabukuro et al., 2024; Thompson et al., 2017). Within Vietnam, prior research has focused mainly on ADHD prevalence, parental awareness, and help‐seeking; no published randomized trial has evaluated a structured BPT program or examined parenting skills as a mechanism for change.

### Study aims and hypotheses

This study evaluated the effectiveness of a 6‐week culturally adapted blended (online group plus home‐based individual support) BPT program for Vietnamese parents of children with ADHD against treatment as usual plus psychoeducation. We hypothesized that, relative to controls, intervention parents would show (a) greater improvements in parenting skills and (b) greater reductions in parent‐rated child ADHD symptoms at post‐intervention and 3‐month follow‐up, and (c) that improvements in parenting skills would mediate intervention effects on child ADHD symptoms. Parenting stress was examined as a secondary outcome variable.

### Conceptual framework

**FIGURE 1:**
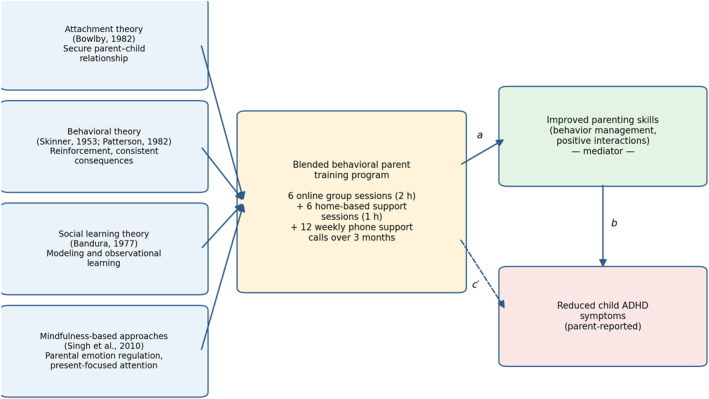
Conceptual framework of blended parenting intervention. Solid arrows denote hypothesized causal paths; *a*, *b*, and *c′* denote the tested mediation paths.

The intervention integrates four theoretical perspectives, with operant behavioral theory and social learning theory as its core, and attachment‐informed and mindfulness‐based components in supporting roles (Figure 1). The behavioral core follows the established BPT tradition: parents learn to modify the antecedents and consequences of child behavior—praise and rewards for adaptive behavior, planned ignoring, and time‐out for maladaptive behavior—per Skinner's (1953) operant principles as applied to coercive family processes by Patterson (1982). Social learning theory (Bandura, 1977) adds modeling: parents are taught that children acquire self‐regulation partly by observing parental behavior, so parents practice modeling organized, planned, and calm responses.

Two supporting components were included because of their expected fit with the Vietnamese context and the emerging evidence. First, an attachment‐informed emphasis on building a secure, positive parent–child relationship (Bowlby, 1982; Bretherton, 1992) was placed at the start of the program (e.g., “special play time”), both because positive parental attention is a prerequisite for effective reinforcement and because local clinicians judged relationship‐building content to be more acceptable to Vietnamese parents as an entry point than immediate discipline techniques. Second, brief mindfulness‐based exercises for parents (breath awareness and body scan) were included to support parental emotion regulation and reduce harsh, reactive discipline; trials of mindfulness‐enhanced BPT and mindful parenting programs reported benefits for parental self‐regulation and discipline practices (Mah et al., 2021; Singh et al., 2010; van der Oord et al., 2012). In the integrated model, all components converge on a single proximal target—parenting skills—which is hypothesized to mediate downstream changes in child ADHD symptoms (Figure 1).

## METHODS

### Design and registration

This was a two‐arm, parallel‐group RCT with partial masking (outcome data collection managed by assessors masked to allocation; participants and facilitators could not be masked) conducted from 2023 to 2025 in an urban region of central Vietnam. Assessments were conducted at baseline (T1), immediately post‐intervention (T2, week 6), and at the 3‐month follow‐up (T3). The study was approved by the Research Ethics Committee of the University of Education, Vietnam National University, Hanoi (approval minutes no. 2023.03/HĐĐĐ‐ĐHGD, dated July 31, 2023) and was conducted within the framework of the funded research project No. B2023‐DN03‐07. The trial was retrospectively registered with the Thai Clinical Trials Registry on February 12, 2026, after study completion (TCTR20260212003); prospective registration was not undertaken at study onset owing to the research team's limited familiarity with trial registration requirements at that time. We acknowledge this transparently and discuss its implications in the limitations section. No a priori power analysis was conducted when the study was planned; a post hoc sensitivity analysis is reported under the Statistical analysis section.

### Participants

#### Recruitment and screening of participants

Recruitment took place over 2 years through the Department of Education in each urban and rural district of the study region. Official invitation packets (invitation letter, protocol description, and link to an online screening survey) were distributed via 49 participating elementary schools. A total of 324 parents completed the online screening, which included the Vanderbilt ADHD Diagnostic Parent Rating Scale (VADPRS) and demographic questions regarding their children. Of these, 147 children (45.4%) screened positive (≥6 symptoms rated “often” or “very often” on the inattention and/or hyperactivity–impulsivity subscales), and their families were invited to a free, in‐person diagnostic assessment at a research clinic, where child psychiatrists and clinical child psychologists confirmed diagnoses and assessed eligibility.

#### Inclusion and exclusion criteria

Children were eligible if they (a) were enrolled in elementary school (approximately 6–11 years); (b) met the DSM‐5 criteria for ADHD (combined or predominantly inattentive presentation), confirmed by a pediatric psychiatrist through a clinical interview with the parent and review of screening ratings, with symptom duration ≥6 months (a standardized semi‐structured diagnostic interview such as the K‐SADS was not available in Vietnamese; we note this as a limitation); (c) had no co‐occurring autism spectrum disorder, intellectual disability, or severe mood or psychotic disorder (children with co‐occurring oppositional or anxiety symptoms below these thresholds were not excluded); and (d) were not currently taking ADHD medication or receiving another behavioral intervention. Parents were eligible if they (a) lived in or near the study area, (b) could commit to the 6‐week program and follow‐up assessments, (c) had basic ability to use online platforms (e.g., Zoom/MS Teams), and (d) did not have a severe mental health condition that would impede participation (operationalized as a self‐reported or documented current diagnosis of a psychotic disorder, bipolar disorder, severe depressive episode, or substance use disorder requiring treatment; parents with milder, managed conditions such as mild depressive or anxiety symptoms were not excluded). We acknowledge that excluding children with major comorbidities and parents with severe mental health conditions limits generalizability, given the high prevalence of comorbidities in ADHD populations (see Limitations).

#### Consent

Consent followed a multi‐step procedure: a research assistant telephoned each screen‐positive family approximately 1 week before the diagnostic visit to explain the study; after eligibility confirmation, a clinical psychologist met each parent individually to detail the program, time commitment, randomization, voluntariness, and data protection; written informed consent (and child assent, where appropriate) was then obtained. Of the 106 eligible families, 20 declined (mostly because of work‐schedule conflicts), leaving 86 parents who consented and completed the baseline assessment.

### Randomization and masking

The 86 consenting participants were randomly allocated 1:1 to the intervention (*n* = 43) or control (*n* = 43) groups. An independent statistician, not involved in recruitment or assessment, generated the allocation using SPSS 26: each participant received a uniform random number (RV.UNIFORM), the list was sorted in ascending order, and the first 43 participants were assigned to the intervention group, with the remaining 43 assigned to the control group. Outcome questionnaires were administered online through coded survey links by staff masked to group allocation; however, because outcomes were parent‐reported and parents knew their allocation, outcome measurement could not be considered masked. Following the current inclusive‐language guidance, we used the term “masking” rather than “blinding” throughout.

### Intervention

#### Structure and rationale for the format

The intervention was a blended program comprising three elements (Table 1): (a) six weekly online group sessions (2 hours each; approximately eight parents per group), delivered via videoconference by two clinical child and adolescent psychologists; (b) six individual home‐based support sessions (1 h each), delivered by trained research assistants within 3–5 days after each group session; and (c) a 3‐month maintenance phase after the sixth session, with weekly support telephone calls (12 calls in total) to reinforce skill use, troubleshoot, and answer questions. This format deliberately differs from the typical 8–12 weekly clinic sessions of 60–90 minutes used in many BPT programs. The condensed six‐session group curriculum reduced the scheduling burden for working parents, while the home‐based sessions individualized the programme by allowing direct observation of parent–child practice—the in vivo feedback function usually served by within‐session rehearsal in longer clinic programmes. Weekly telephone support during follow‐up was added because maintenance of gains after the program ended varies considerably across parenting interventions (van Aar et al., 2017), and because the research team anticipated that newly learned techniques would need support to generalize within multigenerational households. We recognize that this level of continued support exceeds that of most published BPT trials and discuss its implications for interpreting the effects and for implementation (see Discussion).

**TABLE 1:** Intervention program framework.

Session	Main content	Format	Facilitator	Duration
1	Psychoeducation, behavioral principles	Online group	Psychologist	2 h
1—Support	Identify adaptive/maladaptive behaviors, explain causes	In home	Research assistant	1 h
2	Building parent–child relationship, effective instruction	Online group	Psychologist	2 h
2—Support	Observe “special play time,” guide instructions	In home	Research assistant	1 h
3	Reinforcing adaptive behaviors (praise, reward)	Online group	Psychologist	2 h
3—Support	Observe praise techniques, reward planning	In home	Research assistant	1 h
4	Reducing maladaptive behaviors (planned ignoring, time‐out)	Online group	Psychologist	2 h
4—Support	Plan for applying ignoring/time‐out	In home	Research assistant	1 h
5	Mindfulness: Breath awareness, body scan	Online group	Psychologist	2 h
5—Support	Supervised practice, group discussion	In home	Research assistant	1 h
6	Managing behaviors at school and in public	Online group	Psychologist	2 h
6—Support	Discuss real‐life challenges and solutions	In home	Research assistant	1 h

*Note*: Each home‐based support session was delivered within 3–5 days after the corresponding online group session (i.e., in the same week). After the six‐week program, parents received weekly support telephone calls for three months (12 calls) for follow‐up, guidance, and queries.

#### Content

The six group sessions covered: (1) psychoeducation about ADHD and core principles of behavior management; (2) building a positive parent–child relationship and giving effective instructions; (3) strengthening adaptive behavior through praise and rewards; (4) reducing maladaptive behavior using planned ignoring and time‐out; (5) mindfulness practices for parents and children (breath awareness, body scan); and (6) managing behavior at school and in public settings (Table 1; session‐level objectives, activities, skills, and materials are listed in Supporting Information S1 Table S2). “Special play time” was defined as a brief (10–15 minutes), regularly scheduled, child‐directed play period in which the parent follows the child's lead and attends to, describes, and praises the child's appropriate behavior while refraining from commands, questions, and criticism. It serves to strengthen the parent–child relationship and establish parental attention as an effective reinforcer.

#### Home‐based support sessions and the research assistants

Six research assistants, all psychology graduates, delivered the home support sessions (Table 2). Research assistants received structured training: they attended all program training sessions 1 month before delivery, rehearsed home‐support procedures through role‐play under the supervision of clinical psychologists, and were explicitly trained to provide feedback on parents' implementation of techniques with their children (including observing and video‐recording parent–child “special play time” practice and coaching effective instruction delivery). They were instructed to reinforce and troubleshoot only previously taught content and not to introduce any new material. Each session followed a standardized checklist documenting all activities performed, and research assistants attended weekly supervision meetings with supervising clinicians throughout the intervention and follow‐up phases.

**TABLE 2:** Home‐based support session content delivered by research assistants.

Session	Review content	Practical implementation/guidance	Duration
1	Review online session 1 content	List 3 adaptive and 3 maladaptive behaviors; arrange behaviors on the behavioral assessment scale; explain causes of maladaptive behaviors	60 min
2	Review session 1; gather feedback	Observe and video‐record parents implementing special play time; coach specific, effective instructions	60 min
3	Review sessions 1–2; gather feedback	Discuss implementation of praise and reward techniques: Facilitators, barriers, recommendations	60 min
4	Review sessions 1–3; gather feedback	Discuss implementation of planned ignoring and time‐out	60 min
5	Review sessions 1–4; gather feedback	Supervise mindfulness practice; address difficulties	60 min
6	Review sessions 1–5; gather feedback	Discuss managing behavior at school/in public; consolidate plans	60 min

*Note*: Research assistant requirements: attend all training sessions 1 month before the program; rehearse home support delivery via role‐play under clinical supervision; reinforce only previously taught content; complete a standardized checklist after each session; record a proportion of sessions for supervision review (achieved: ≈40%); attend weekly supervision meetings.

#### Treatment fidelity, attendance, and compliance were assessed

Fidelity was supported by a facilitator's manual, standardized session checklists, and weekly supervision of facilitators and research assistants. Per protocol, a proportion of home support sessions were audio/video recorded for supervision review; in practice, approximately 40% of sessions were recorded and reviewed, exceeding the planned 20%. Checklist completion was required after each session. Attendance among the 37 intervention completers was 100% for the six online group sessions and six home‐based support sessions; the three participants who discontinued before T2 did so during the program (their session‐level attendance records were not retained). Weekly support calls during the maintenance phase were completed as scheduled for all the completers.

#### Control condition

Control participants received treatment as usual plus psychoeducation: at allocation (immediately after randomization), they were given educational books and printed materials on ADHD and its management. No restrictions were placed on services that families might seek in the community; community ADHD services in the study region were very limited, and the use of additional services during the trial was not systematically monitored—a limitation we acknowledge. Therefore, the condition is best characterized as a low‐intensity psychoeducational comparison rather than a pure waiting list. Control families were offered the full intervention program after the 3‐month follow‐up.

### Cultural adaptation

The program was newly developed from the international BPT evidence base and was systematically adapted for Vietnamese families. The adaptation process drew on (a) a local pre‐intervention survey of parents of children with ADHD in the study region, covering parental knowledge of ADHD, parenting skills, parent–child relationship quality, parenting stress, and attitudes toward parent training (findings summarized in Supporting Information S1 Table S1); (b) input from Vietnamese clinical child psychologists and a pediatric psychiatrist during program development; and (c) piloting of materials with parents for comprehensibility of the materials. The concrete adaptations included the following: First, language and materials: all content was written in plain Vietnamese, technical terms were simplified (e.g., “củng cố tích cực” [positive reinforcement] was introduced through everyday examples), and all handouts, worksheets, and illustrative videos were newly produced with Vietnamese actors and family settings. Second, culturally anchored examples: praise and reward examples were built around homework completion and school routines—the dominant arena of parent–child conflict in this context; time‐out and planned‐ignoring scripts were adjusted for households where grandparents co‐resided, including guidance for enlisting grandparents' cooperation so that ignoring is not undermined by another adult attending to the behavior. Third, normalizing praise: Because overt praise of children is less normative in Vietnamese families and some parents feared that praise would “spoil” the child, psychoeducation directly addressed this belief, framing labeled praise as consistent with the parental duty to “train” the child (Chao, 1994; Mestechkina et al., 2014). Fourth, content sequencing: relationship‐building content was placed before discipline techniques to align with the cultural priority of family harmony. Finally, the lessons were reorganized to reduce redundancy and increase practice exercises based on the local survey results. The shift from face‐to‐face to online delivery with instructional videos was a delivery‐mode modification for accessibility rather than a cultural adaptation.

### Measures

#### Parenting skills (primary outcome)

The Parenting Skills Assessment Scale [PSAS] for ADHD was developed for this study based on existing parenting questionnaires and was tailored to ADHD contexts. It presents 11 common parent–child situations (e.g., the child refuses to do homework; the child acts out in public). For each situation, parents rated 7–10 possible responses—both recommended strategies (e.g., labeled praise, calm rule‐setting) and ineffective strategies (e.g., yelling, giving up)—on frequency of use (0 = never to 2 = frequently) and perceived effectiveness (0 = not effective to 2 = very effective). The composite score sums the frequency and effectiveness ratings across situations, with higher scores indicating more frequent and effective use of the recommended strategies. The internal consistency in this sample was good (Cronbach's *α* = 0.87). The PSAS was administered at T1, T2, and T3 time points.

#### Child ADHD symptoms (primary outcome)

ADHD symptom severity was assessed using the VADPRS (Bard et al., 2013; Wolraich et al., 2003), using the 18 DSM‐based symptom items (nine inattention, nine hyperactivity/impulsivity), each rated from 0 (never) to 3 (very often); the total symptom score ranges from 0 to 54. The VADPRS has well‐documented reliability and validity (Bard et al., 2013). In this sample, *α* = 0.89 (inattention) and 0.90 (hyperactivity/impulsivity). The performance/impairment items of the Vanderbilt were not administered; therefore, functional impairment could not be analyzed as an outcome (see limitations). Symptom ratings were obtained at T1, T2, and T3 time points.

#### Parenting stress (secondary outcome variable)

Parenting stress related to child behavior was measured using a 10‐scenario questionnaire adapted from the Disruptive Behavior Stress Inventory (Johnson & Reader, 2002). Parents rated their stress reaction to each common difficult child behavior scenario (e.g., tantrum in a public place) from 0 (not at all stressful) to three (extremely stressful); *α* = 0.84. Stress was measured at T1, T2, and T3 time points. Consistent with the trial registration, parenting stress is reported here as a secondary outcome and was used as a covariate in a sensitivity mediation model.

#### Program acceptability and feasibility

Intervention parents completed a short program‐perception survey (perceived usefulness of each session, ease of implementing strategies, barriers; *α* = 0.70) and a debriefing interview at follow‐up. Key acceptability results are summarized in the Results section (with details in the Supporting Information S1 Table S1), as they inform the interpretation of the quantitative findings.

### Statistical analysis

Analyses were conducted using SPSS 26 (with PROCESS‐equivalent mediation models verified in Python/statsmodels). A two‐tailed *α* = 0.05 was used. The baseline characteristics are presented descriptively by group. Following the methodological guidance and the CONSORT statement, no significance tests of baseline differences were conducted (de Boer et al., 2015; Schulz et al., 2010).

#### Analytical population and missing data

**FIGURE 2:**
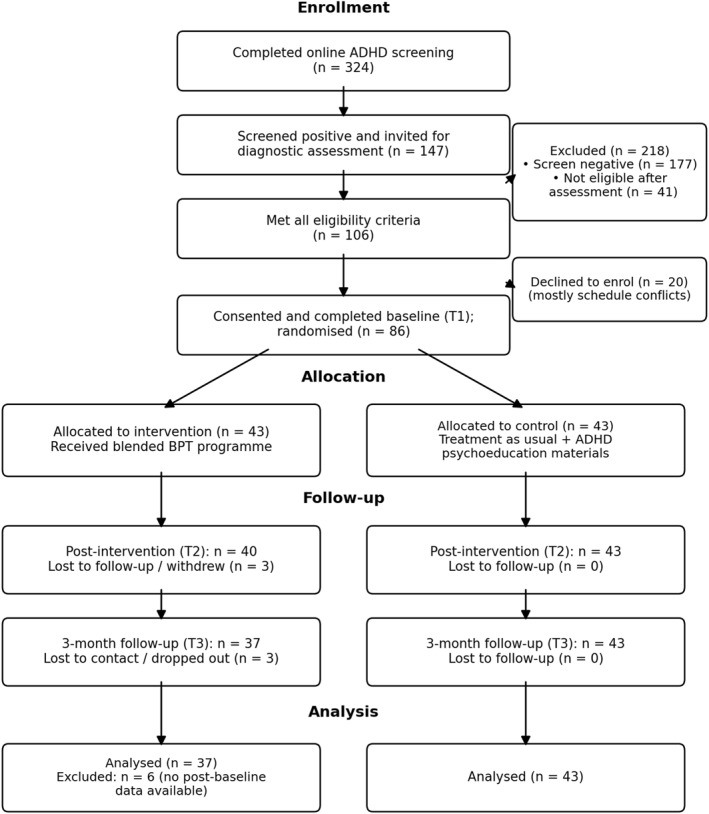
CONSORT (consolidated standards of reporting trials) flow diagram.

All 86 randomized participants were accounted for in the CONSORT diagram (Figure 2). Six intervention participants discontinued (three before T2; three more before T3) and did not complete outcome assessments after baseline; their post‐baseline data were unavailable and their baseline records were not retained in the analysis database, so dropout‐versus‐completer baseline comparisons and multiple imputation could not be performed. Therefore, the analyses used the completer population (*N* = 80: intervention, *n* = 37; control, *n* = 43; 93% of those randomized). We report this transparently as a deviation from the full intention‐to‐treat analysis and consider its implications for bias in the Limitations section. No interim analyses were performed.

#### Primary outcome analysis

For each outcome (PSAS, ADHD symptoms, parenting stress), we estimated a 2 (group) × 3 (time: T1, T2, T3) mixed‐design ANOVA, applying Greenhouse–Geisser correction where sphericity was violated, with the group × time interaction as the effect of interest. Because mixed ANOVA on completers does not adjust for baseline values, the primary between‐group tests were analyses of covariance (ANCOVA) on T2 and T3 scores with the baseline score as a covariate, which is the recommended approach for randomized pre‐post designs. Effect sizes were reported as partial *η* ^*2*^ and baseline‐adjusted Cohen's *d* (adjusted mean difference divided by pooled SD). Within‐group changes were examined using paired *t‐tests*.

#### Mediation analyses

The pre‐specified mechanism model (Hayes, 2022, Model 4 logic) used group (0 = control, 1 = intervention) as the independent variable, change in parenting skills from T1 to T2 as the mediator, and T3 ADHD symptoms as the outcome, while controlling for T1 ADHD symptoms. This specification preserves temporal ordering (mediator change precedes outcome measurement). Indirect effects were tested using 5000 bootstrap resamples and 95% confidence intervals. Two sensitivity models were estimated: (a) T1→T3 change scores for both the mediator and outcome, and (b) the primary model additionally adjusting for T3 parenting stress to examine the specificity of the parenting skills pathway. Given the modest sample size and the absence of a significant total between‐group effect on ADHD symptoms, mediation analyses are presented as exploratory mechanism evidence rather than confirmatory tests.

#### Nesting and power

The intervention participants were nested within training groups of approximately eight parents, facilitated by the same two psychologists. With few groups, multilevel modeling was not feasible, and we acknowledge the possible underestimation of standard errors for the intervention arm effects. No a priori power analyses were conducted. A post hoc sensitivity analysis indicated that with *n* = 37 and *n* = 43 per arm, two‐tailed *α* = 0.05, and 80% power, the smallest detectable between‐group effect was *d* ≈ 0.63 for an unadjusted comparison (smaller for baseline‐adjusted ANCOVA); thus, the trial was adequately powered for the large parenting‐skills effect but underpowered for small effects on ADHD symptoms.

## RESULTS

### Participant flow and characteristics

Figure 2 shows the CONSORT flow diagram. Of the 86 randomized parents, 80 (93%) completed all three assessments and were analyzed (intervention: 37/43, 86%; control: 43/43, 100%).

Table 3 presents the baseline characteristics descriptively by group, per CONSORT guidance (de Boer et al., 2015). Caregivers were predominantly mothers (86%), most parents were 20–45 years old, most families had two children, and approximately one‐third lived in multigenerational households with grandparents. Most children were boys (63.7% overall), with a mean age of approximately 8.5 years; about two‐thirds had combined presentation, and one‐third had predominantly inattentive presentation, distributed similarly across groups. Baseline ADHD symptom scores (intervention: *M* = 21.05, SD = 8.91; control: *M* = 19.58, SD = 7.90) and parenting skills (intervention: *M* = 261.14, SD = 36.03; control: *M* = 264.35, SD = 30.82) were similar across the groups. Descriptively, intervention group parents were somewhat more often employed in administrative/service occupations, and control group parents were more often in professional/managerial or business occupations.

**TABLE 3:** Baseline characteristics of the analyzed sample (descriptive only).

Characteristic	Intervention (*n* = 37)	Control (*n* = 43)
Parents		
Age 20–35 years, *n* (%)	18 (48.6)	18 (41.9)
Age 36–45 years, *n* (%)	19 (51.4)	23 (53.5)
Age >45 years, *n* (%)	0 (0.0)	2 (4.7)
Mother as participating caregiver, *n* (%)	35 (94.6)	34 (79.1)
Occupation: Administrative/service employee, *n* (%)	23 (62.2)	18 (41.9)
Occupation: Professional/manager, *n* (%)	6 (16.2)	13 (30.2)
Occupation: Business/trade, *n* (%)	5 (13.5)	12 (27.9)
Occupation: Self‐employed/freelance, *n* (%)	3 (8.1)	0 (0.0)
Married, *n* (%)	33 (89.2)	37 (86.0)
Single parent, *n* (%)	1 (2.7)	2 (4.7)
Separated/divorced, *n* (%)	3 (8.1)	4 (9.3)
Income <7 million VND/month, *n* (%)	9 (24.3)	7 (16.3)
Income 7–15 million VND/month, *n* (%)	16 (43.2)	17 (39.5)
Income 15–25 million VND/month, *n* (%)	8 (21.6)	16 (37.2)
Income >25 million VND/month, *n* (%)	4 (10.8)	3 (7.0)
Living as nuclear family, *n* (%)	22 (59.5)	27 (62.8)
Living with grandparents, *n* (%)	11 (29.7)	15 (34.9)
Living with other relatives, *n* (%)	4 (10.8)	1 (2.3)
Family		
One child, *n* (%)	5 (13.5)	8 (18.6)
Two children, *n* (%)	27 (73.0)	29 (67.4)
Three or more children, *n* (%)	5 (13.5)	6 (14.0)
Children		
Boys, *n* (%)	27 (73.0)	24 (55.8)
Child baseline scores, *M* (SD)		
Vanderbilt inattention subscale	12.35 (4.80)	10.91 (4.13)
Vanderbilt hyperactivity/impulsivity subscale	8.70 (5.22)	8.67 (4.83)
Vanderbilt total ADHD symptoms	21.05 (8.91)	19.58 (7.90)
Parent baseline scores, *M* (SD)		
Parenting skills (PSAS)	261.14 (36.03)	264.35 (30.82)
Parenting stress	14.41 (17.12)	12.77 (12.61)

*Note*: Following the CONSORT guidelines, baseline differences were not tested statistically (de Boer et al., 2015).

Abbreviations: PSAS, Parenting Skills Assessment Scale; VND, Vietnamese dong.

### Parenting skills

The descriptive statistics for both groups at all time points are presented in Table 4. The 2 × 3 mixed ANOVA showed a significant group × time interaction, *F* (2, 156) = 20.75, *p* < .001, partial *η* ^*2*^ = 0.21 (sphericity was violated, Mauchly's *W* = 0.78, *p* < .001; the interaction remained significant with Greenhouse–Geisser correction *ε* = 0.82). The main effects of time, *F* (2, 156) = 32.62, *p* < .001, partial *η* ^*2*^ = 0.29, and group, *F* (1, 78) = 18.73, *p* < .001, partial *η* ^*2*^ = 0.19, were qualified by this interaction.

**TABLE 4:** Parenting skills (PSAS) by group and time, with between‐group ANCOVA results.

Time	Group	*M* (SD)	Within‐group change from T1	Baseline‐adjusted between‐group difference
T1	Intervention	261.14 (36.03)	–	–
T1	Control	264.35 (30.82)	–	–
T2	Intervention	313.70 (40.03)	+52.57, *p* < .001	45.01 (SE 8.37), *F* (1, 77) = 28.90, *p* < .001, *η* ^*2*^_*p*_ = 0.27, *d* = 1.17
T2	Control	269.74 (37.35)	+5.40, *p* = .225	
T3	Intervention	315.16 (28.87)	+54.03, *p* < .001	43.05 (SE 6.59), *F* (1, 77) = 42.67, *p* < .001, *η* ^*2*^_*p*_ = 0.36, *d* = 1.28
T3	Control	273.74 (37.37)	+9.40, *p* = .031	

*Note*: *N* = 80. Group × time interaction: *F* (2, 156) = 20.75, *p* < .001, partial *η* ^*2*^ = 0.21. Adjusted *d* = adjusted mean difference divided by the pooled SD at that time point.

Baseline‐adjusted ANCOVAs confirmed large between‐group differences at both post‐treatment assessments: at T2, adjusted mean difference = 45.01 points (SE = 8.37), *F* (1, 77) = 28.90, *p* < .001, partial *η* ^*2*^ = 0.27, adjusted *d* = 1.17; at T3, adjusted mean difference = 43.05 points (SE = 6.59), *F* (1, 77) = 42.67, *p* < .001, partial *η* ^*2*^ = 0.36, adjusted *d* = 1.28.

Within the intervention group, parenting skills increased sharply from T1 to T2 (Δ*M* = 52.57, *t* (36) = 5.74, *p* < .001, *d*_*z*_ = 0.94) and remained stable from T2 to T3 (Δ*M* = 1.46, *p* = .786), yielding a sustained T1→T3 gain (Δ*M* = 54.03, *t* (36) = 8.38, *p* < .001, *d*_*z*_ = 1.38). Within the control group, the T1→T2 change was not significant (Δ*M* = 5.40, *t* (42) = 1.23, *p* = .225); a small cumulative increase emerged at T3 (Δ*M* = 9.40, *t* (42) = 2.23, *p* = .031, *d*_*z*_ = 0.34).

### Child ADHD symptoms

The descriptive statistics are presented in Table 5. The group × time interaction in the mixed ANOVA did not reach statistical significance (*F* (2, 156) = 2.71, *p* = .070, partial *η* ^2^ = 0.03). There was a significant main effect of time, *F* (2, 156) = 6.93, *p* = .001 (Greenhouse–Geisser corrected *p* = .002), partial *η* ^*2*^ = 0.08, and no main effect of group, *F* (1, 78) = 0.06, *p* = .806.

**TABLE 5:** Parent‐rated ADHD symptoms (VADPRS) by group and time, with between‐group ANCOVA results.

Time	Group	*M* (SD)	Within‐group change from T1	Baseline‐adjusted between‐group difference
T1	Intervention	21.05 (8.91)	–	–
T1	Control	19.58 (7.90)	–	–
T2	Intervention	17.84 (8.72)	−3.22, *p* = .003	−2.38 (SE 1.23), *F* (1, 77) = 3.73, *p* = .057, *η* ^*2*^_*p*_ = 0.05, *d* = 0.28
T2	Control	19.07 (8.26)	−0.51, *p* = .541	
T3	Intervention	16.92 (8.95)	−4.14, *p* < .001	−2.42 (SE 1.34), *F* (1, 77) = 3.26, *p* = .075, *η* ^*2*^_*p*_ = 0.04, *d* = 0.30
T3	Control	18.40 (7.11)	−1.19, *p* = .232	

*Note*: *N* = 80. Group × time interaction: *F* (2, 156) = 2.71, *p* = .070, partial *η* ^*2*^ = 0.03.

Abbreviation: VADPRS, Vanderbilt ADHD Diagnostic Parent Rating Scale (symptom score range, 0–54).

Baseline‐adjusted ANCOVAs showed small between‐group differences favoring the intervention that did not reach statistical significance: at T2, adjusted mean difference = −2.38 points (SE = 1.23), *F* (1, 77) = 3.73, *p* = .057, partial *η* ^*2*^ = .05, adjusted *d* = 0.28; at T3, adjusted mean difference = −2.42 points (SE = 1.34), *F* (1, 77) = 3.26, *p* = .075, partial *η* ^*2*^ = .04, adjusted *d* = 0.30 (unadjusted *d* = 0.15 and 0.18, respectively).

Within the intervention group, parent‐rated ADHD symptoms decreased significantly from T1 to T2 (Δ*M* = −3.22, *t*(36) = 3.23, *p* = .003, *d*_*z*_ = 0.53) and from T1 to T3 (Δ*M* = −4.14, *t*(36) = 3.65, *p* < .001, *d*_*z*_ = 0.60), with no significant additional change from T2 to T3 (*p* = .314). Within the control group, no changes were significant (T1→T2: Δ*M* = −0.51, *p* = .541; T1→T3: Δ*M* = −1.19, *p* = .232). In absolute terms, a reduction of three to four points on the 0–54 Vanderbilt symptom scale is unlikely to change the diagnostic status, although it may reflect perceptible improvements in specific behaviors.

### Parenting stress (secondary outcome)

Parenting stress declined over time across the whole sample, main effect of time *F* (2, 156) = 7.38, *p* = .001, partial *η* ^*2*^ = 0.09, with no group × time interaction, *F* (2, 156) = 0.21, *p* = .809, and no group main effect, *F* (1, 78) = 0.31, *p* = .580 (Table 6). Baseline‐adjusted between‐group differences were not significant at T2 (*p* = .994) or T3 (*p* = .648). Thus, the intervention did not reduce stress beyond that observed in the control condition.

**TABLE 6:** Parenting stress by group and time.

Time	Intervention *M* (SD)	Control *M* (SD)
T1	14.41 (17.12)	12.77 (12.61)
T2	10.27 (11.70)	9.37 (12.04)
T3	11.35 (18.31)	9.05 (12.11)

*Note*: *N* = 80. Main effect of time *p* = .001; group × time interaction *p* = .809.

### Association between parenting skills and ADHD symptoms

Across the full sample at T3, parenting skills were negatively correlated with ADHD symptoms, *r* (78) = −0.41, *p* < .001. In a simple linear regression, parenting skills significantly predicted ADHD symptom severity, *B* = −0.083, *β* = −0.41, *t* (78) = −3.96, *p* < .001, *R* ^*2*^ = 0.17; a 10‐point higher PSAS score was associated with approximately 0.8 points lower symptom score.

### Exploratory mediation analyses

Table 7 summarizes the mediation model. In the pre‐specified model (group → T1–T2 change in parenting skills → T3 ADHD symptoms, controlling for T1 symptoms), the intervention strongly predicted skill gains (*a* = 46.22, SE = 9.74, *p* < .001), and skill gains predicted lower follow‐up symptoms (*b* = −0.040, SE = 0.015, *p* = .010). The indirect effect was significant, *ab* = −1.86, bootstrap SE = 0.93, 95% CI [−4.01, −0.37], whereas the direct effect was not (*c′* = −0.56, *p* = .702). Notably, the total effect of the group on T3 symptoms was not statistically significant (*c* = −2.42, *p* = .075); the mediation findings therefore indicate a significant indirect pathway rather than full mediation of an established total effect and should be interpreted as exploratory mechanism evidence.

**TABLE 7:** Mediation analyses: Parenting skills as mediator of the intervention–ADHD association.

Path/effect	B	SE	*p*	95% CI
Primary model (*M* = Δ PSAS T1→T2; Y = T3 ADHD, controlling T1 ADHD)				
Total effect: Group → ADHD (c)	−2.42	1.34	.075	[−5.09, 0.25]
a: Group → Δ parenting skills	46.22	9.74	<.001	[26.83, 65.61]
b: Δ parenting skills → ADHD	−0.040	0.015	.010	[−.070, −.010]
Direct effect (c′)	−0.56	1.47	.702	[−3.49, 2.37]
Indirect effect (a × b)^a^	−1.86	0.93	–	[−4.01, −0.37]
Sensitivity 1 (*M* = Δ PSAS T1→T3; Y = Δ ADHD T1→T3)				
Indirect effect (a × b)^a^	−4.44	1.48	–	[−7.78, −2.03]
Sensitivity 2 (primary model + T3 parenting stress covariate)				
Indirect effect (a × b)^a^	−1.67	0.78	–	[−3.47, −0.45]

*Note*: *N* = 80.

^a^ Bootstrap standard errors and percentile confidence intervals based on 5000 resamples; an indirect effect was considered statistically significant when the 95% CI excluded zero.

Sensitivity analyses yielded the same pattern: using T1→T3 change scores for mediator and outcome, *ab* = −4.44, 95% CI [−7.78, −2.03]; additionally adjusting the primary model for T3 parenting stress, *ab* = −1.67, 95% CI [−3.47, −0.45], with parenting stress positively associated with symptoms (*p* < .001) but not accounting for the skills pathway. The specificity of the indirect effect on parenting skills, together with the temporal ordering of the mediator and outcome, is consistent with the hypothesized mechanism, although causal interpretation requires larger confirmatory trials.

### Acceptability and feasibility

Among intervention completers, attendance was 100% for both online group and home‐based support sessions, and all scheduled weekly maintenance calls were completed. On the program‐perception survey, parents rated the program's usefulness and feasibility highly, and debrief interviews indicated high satisfaction with the flexibility of the online format and the practical value of home‐based coaching, with suggested improvements centered on session scheduling and more video examples. The full acceptability data are presented in the Supporting Information S1 Table S2.

## DISCUSSION

This RCT evaluated a culturally adapted blended BPT program for Vietnamese parents of elementary school‐aged children with ADHD. Three findings stand out in this study. First, the program produced a large, statistically robust, and durable improvement in parenting skills (baseline‐adjusted *d* = 1.17 at post‐intervention and 1.28 at the 3‐month follow‐up). Second, the effects on parent‐rated child ADHD symptoms were small and did not reach statistical significance in between‐group analyses (adjusted *d* ≈ 0.28–0.30, *p* = .057–.075), although symptoms declined significantly within the intervention group and not within the control group. Third, exploratory mediation analyses showed a significant indirect pathway from the intervention through improved parenting skills to lower follow‐up ADHD symptoms, consistent with the theorized mechanism of change in the study. However, in the absence of a significant total effect, this constitutes preliminary mechanistic evidence rather than a demonstration of full mediation.

### Parenting skills

The magnitude of the parenting‐skills effect exceeds the small‐to‐medium pooled effects on parenting outcomes reported in meta‐analyses of BPT (Daley et al., 2014; Dekkers et al., 2022; Kaminski et al., 2008; Sanders et al., 2014). Several explanations deserve consideration in order of their importance. First, and most plausibly, the intensity and structure of support in this trial were unusual: in addition to six group sessions, every parent received six individual home‐based coaching sessions with direct observation and feedback on technique implementation, followed by 12 weekly support telephone calls during the maintenance period. Few BPT trials in the existing meta‐analytic literature include any comparable level of individualized, home‐based, post‐program support; this design feature, rather than cultural context, is the most parsimonious explanation for the large skills effect and has direct implications for practitioners (see Clinical implications). Second, measurement factors likely contributed: the PSAS is a self‐report instrument developed for this program and closely aligned with its content, and parents who have just completed training may rate their own skills more favorably (a training‐exposure reporting bias), inflating effects relative to observational measures. Third, cultural and motivational factors may have played a supporting role: in Confucian‐heritage contexts where the “training” of children is a core parental duty (Chao, 1994), the motivation to acquire and practice concrete techniques may be high, and the free, accessible online format with modest compensation may have supported engagement. These explanations are not mutually exclusive, and the 100% session attendance is consistent with high engagement.

### Child ADHD symptoms

The small, non‐significant between‐group effect on parent‐rated ADHD symptoms is consistent with broader literature. Meta‐analyses indicate that behavioral interventions yield small parent‐rated improvements in ADHD symptoms (e.g., SMD ≈ 0.35 for most proximal raters) that attenuate toward zero with probably masked assessors (Corcoran & Dattalo, 2006; Daley et al., 2014). The neurodevelopmental basis of ADHD—heritability of approximately 74% (Faraone & Larsson, 2019) and replicated subcortical structural differences (Hoogman et al., 2017)—sets realistic boundaries on how much core symptom change parent training alone can produce. BPT's strongest and most reliable effects are on parenting behavior, parent–child interaction, child oppositional/conduct problems, and functional outcomes rather than core symptoms (Daley et al., 2014; Dekkers et al., 2022; Nobel et al., 2020). Because our trial did not administer the Vanderbilt performance/impairment items, we could not test whether the program improved functional impairment, which recent individual‐participant‐data evidence identifies as a key outcome influenced by parenting change (Psyllou et al., 2025), and this is an important target for future studies. Our within‐group symptom reduction (≈4 points) accompanied by marginal between‐group differences also illustrates why within‐group changes in uncontrolled studies should not be read as intervention effects.

### Mechanisms

The significant indirect effect through parenting skills—robust across two alternative specifications and after adjustment for parenting stress—aligns with mediation evidence from Western samples (Forehand et al., 2014; Psyllou et al., 2025) and supports the program's theory of change: training alters parenting practices, and altered parenting practices are associated with subsequent reductions in parent‐rated symptoms. The specificity of the pathway (parenting skills, not parenting stress) and the temporal ordering of the mediator (T1 → T2 change) and outcome (T3) strengthen this interpretation. Nevertheless, several cautions apply: the total effect on symptoms was not significant; both the mediator and outcome were parent‐reported (shared method variance can inflate the *b* path); parenting–ADHD associations are known to be bidirectional, with child symptoms also shaping parenting over time (Shelleby & Ogg, 2020); and the modest sample limits the power for multivariable mechanism models. Therefore, pr, weesent these analyses as exploratory mechanism evidence to be confirmed in adequately powered trials, ideally with multi‐informant or observational measures of both parenting and child behavior.

The absence of a differential effect on parenting stress—stress declined in both arms—contrasts with hybrid programs that explicitly target parental well‐being and have shown stress effects (Shimabukuro et al., 2024 [insert reference]). Our program contained only brief mindfulness elements for parents; more substantial well‐being content may be needed to improve stress outcomes in this population.

### Strengths and limitations

Strengths of the study include the randomized controlled design with a 3‐month follow‐up, systematic stakeholder‐informed cultural adaptation, blended delivery model with documented fidelity procedures (standardized checklists, approximately 40% of home sessions recorded and reviewed, weekly supervision), complete attendance among completers, outcome data collection administered by masked staff, and mechanism‐oriented analyses with sensitivity checks.

These limitations are substantial and constrain causal and generalizability claims. First, analyses were restricted to completers (80/86; 93%): the six intervention dropouts provided no post‐baseline data, and their baseline records were not retained in the analysis database, precluding intention‐to‐treat analyses, multiple imputation, and dropout‐versus‐completer comparisons. Because attrition occurred only in the intervention arm, the effects on parenting skills could be overestimated if parents who benefited less were more likely to discontinue. Second, the trial was registered retrospectively after data collection; although the analyses reported follow the measures collected, retrospective registration weakens protection against selective reporting, and the registration's planned sample (*N* = 90) was specified without an a priori power analysis. Third, all outcomes were parent‐reported by unmasked participants; expectancy and social desirability biases, which may be stronger in the intervention arm, cannot be excluded, and no teacher reports, clinician ratings, or direct observations were collected for comparison. Fourth, ADHD diagnoses were confirmed by clinical interviews rather than a standardized semi‐structured diagnostic instrument. Fifth, functional impairment was not assessed. Sixth, the exclusion of children with major psychiatric comorbidities and parents with severe mental health conditions, combined with a predominantly urban, relatively educated sample, limits generalizability to routine clinical populations, in which comorbidity is the norm. Seventh, the control group's service use was not systematically monitored. Eighth, the intervention participants were nested within the training groups, and our analyses could not model this dependency. Finally, although the PSAS is internally consistent, it is a newly developed self‐report measure, and its validity against observed parenting remains to be established.

### Clinical implications and future directions

Two implications follow directly from these findings. First, for providers in Vietnam and comparable settings, a blended model (brief online group training combined with individual home‐based coaching and structured telephone follow‐up) is feasible, highly acceptable, and effective for building parenting skills. Home‐based feedback sessions and maintenance calls are the program's most distinctive features and warrant evaluation as active ingredients in dismantling designs. Second, claims about child outcomes should remain modest: based on current evidence, this program reliably changes parenting, while its effects on core ADHD symptoms are small and statistically uncertain. Future research should employ a priori powered, prospectively registered trials with intention‐to‐treat analyses; include masked, multi‐informant outcomes (teacher ratings, observation) and functional impairment measures; test maintenance beyond 3 months; examine moderators (child age, sex, severity, comorbidity) and the contribution of grandparents as co‐caregivers; and evaluate whether cultural adaptation itself adds benefit, comparing culturally adapted with non‐adapted versions directly.

## CONCLUSION

In the first RCT of a culturally adapted blended BPT program for parents of children with ADHD in Vietnam, the program produced large, sustained improvements in parenting skills and was delivered with high fidelity and complete attendance. The effects on parent‐rated ADHD symptoms were small and not statistically significant between groups, and parenting stress improved equally in both arms. Exploratory analyses supported the program's theory of change, with improved parenting skills mediating the association between the intervention and follow‐up symptoms. These results position parent training as a feasible, scalable strategy for strengthening parenting capacity in lower‐resource Asian contexts, while underscoring that conclusions about symptom‐level benefits await larger, prospectively registered trials with masked, multi‐informant outcomes.

## AUTHOR CONTRIBUTIONS

**Tran Thanh Nam**: Conceptualization; methodology; formal analysis; supervision; writing—original draft; writing—review and editing. **Nguyen Thi Tram Anh**: Conceptualization; methodology; supervision; writing—review and editing. **Nguyen Thi Hong Nhung**: Conceptualization; investigation; data curation; formal analysis; funding acquisition; project administration; visualization; writing—original draft; writing—review and editing. **Le Thi Lam**: Investigation; data curation; writing—review and editing. **Le Thi Thuy Hang**: Resources; investigation; writing—original draft.

## CONFLICT OF INTEREST STATEMENT

The authors declare no conflicts of interest.

## ETHICAL CONSIDERATIONS

Written informed consent was obtained from all parents/legal guardians prior to their children's participation. Children aged 7 years and older provided verbal assent. This study was approved by the Ethics Committee of the University of Education, Vietnam National University, Hanoi on July 31, 2023 (No. 2023.06.007).

## Supporting information

Supporting Information S1

## Data Availability

The data that support the findings of this study are available from the corresponding author upon reasonable request.
