# Supplementary material for: Effectiveness of a blended online behavioral parent training program for Vietnamese parents of children with ADHD: A randomized controlled trial with 3‐month follow‐up
Source: JCPP Adv. 2026 Jul 24:e70150. Online ahead of print. doi: 10.1002/jcv2.70150 (PMC13400807; doi:10.1002/jcv2.70150)
Supplement: Supplementary file 1 — Supporting Information S1 [file JCV2-9999-e70150-s001.docx]

**Effectiveness of a Blended Online Behavioral Parent Training Program for Vietnamese Parents of Children with ADHD: A Randomized Controlled Trial with 3-Month Follow-Up**

**Supporting Information**

**Table S1.** Framework for developing the parenting behavior training program for parents of children with ADHD

| Intervention component | Theoretical basis | Local survey findings | Identified gaps | Program solutions |
| --- | --- | --- | --- | --- |
| 1. Psychoeducation for parents | Parents of children with ADHD often attribute behavior to internal causes and perceive limited control; many struggle to accept the diagnosis and self-blame | 85% of parents had low-to-moderate awareness of ADHD | Parents lack adequate knowledge about ADHD and how to support their children | Enhance ADHD knowledge, strengthen motivation, explain causes of behavioral problems and supportive strategies |
| 2. Parent–child relationship | Attachment theory (Bowlby, 1982; Bretherton, 1992): caregiver sensitivity fosters secure attachment; parent–child relationships are often disrupted in ADHD | More than 80% of parent–child relationships rated as moderate or poor | Parent–child relationships require improvement | Teach “special play time” and effective instruction to enhance bonding |
| 3. Child behavior management | Behavioral theory (Patterson, 1982; Skinner, 1953): behavior maintained by consequences; social learning theory (Bandura, 1977): children learn through observation | 17.5% of parents had low parenting skills; 70% moderate | Behavior-management skills mostly low to moderate | Train reinforcement of adaptive behavior (praise, rewards) and reduction of maladaptive behavior (planned ignoring, time-out) |
| 4. Stress reduction and symptom support | Parenting stress is elevated in ADHD and correlates with severity (Theule et al., 2013); family mindfulness interventions can reduce parental stress | 97.5% of parents had experienced at least one stressful child-behavior situation; stress higher for combined presentation (M = 16.7) than inattentive (M = 6.4) | High caregiving-related stress; children need attention support | Teach parents mindfulness practices (breath awareness, body scan) |
| 5. Managing behavior at school and in public | Behavioral parent training and classroom contingency management are evidence-based; contingency principles | Public-setting and school-setting situations showed the lowest skill levels in the local survey | Skill gap for school/public settings across the community | Guidance on managing behavior in school/public settings and communicating with teachers |

**Table S2.** Detailed summary of the six-session parent training program

| Session | Main objectives | Core activities | Parent skills acquired | Tools/Materials |
| --- | --- | --- | --- | --- |
| 1. Psychoeducation and core principles | Understand ADHD, child behaviors, and core principles of parent training | Program introduction; discussion on ADHD; four-factor behavior model; observation tools | Identify adaptive vs. maladaptive behaviors; analyze behavioral causes | Slides, four-factor model handout, behavior observation sheets |
| 2. Building positive parent–child relationship and effective instructions | Strengthen parent–child bond; give effective instructions | Homework review; “special play time”; practicing effective instructions | Positive play skills; clear, concise instruction-giving | Demonstration videos, observation sheets, homework |
| 3. Strengthening adaptive behaviors | Increase desirable behaviors using praise and rewards | Homework review; praising techniques; building reward systems | Labeled praise; designing and applying a reward system | Instructional materials, sample reward charts, homework |
| 4. Reducing maladaptive behaviors | Reduce unwanted behaviors using planned ignoring and time-out | Homework review; practicing planned ignoring; practicing time-out | Planned ignoring; correct application of time-out | Step-by-step guides, demonstration videos, homework |
| 5. Mindfulness practice | Reduce parental stress; support symptom management | Homework review; guided breath awareness; body-scan exercise | Relaxation and stress-reduction skills; practicing mindfulness with children | Demonstration videos, self-monitoring worksheets, homework |
| 6. Managing behavior in school and public settings | Support behavior management at school and in public | Homework review; real-life case discussion; practicing teacher collaboration | Communicating with teachers; managing behavior in public | Teacher-collaboration guidelines, case worksheets, homework |
